# High sensitivity blood-based M-protein detection in sCR patients with multiple myeloma

**DOI:** 10.1038/bcj.2017.75

**Published:** 2017-08-25

**Authors:** J R Mills, D R Barnidge, A Dispenzieri, D L Murray

**Affiliations:** 1 Protein Immunology Laboratory, Department of Laboratory Medicine and Pathology, Mayo Clinic, Rochester, MN, USA; 2 Division of Hematology, Department of Medicine, Mayo Clinic, Rochester, MN, USA

## Abstract

We assessed the ability of a mass spectrometry-based technique, called monoclonal immunoglobulin rapid accurate mass measurement (miRAMM), to extend the analytical range of M-protein detection in serum samples obtained from myeloma patients in stringent complete response (sCR) post-autologous stem cell transplant (ASCT). To aid the M-protein detection post ASCT, the accurate molecular mass of the M-protein light chain at diagnosis was determined in all patients (*N*=30) and used to positively identify clones in the sCR serum. Day 100 post-ASCT, sCR samples had miRAMM identifiable M-proteins in 81% of patients. Patients who had achieved only a partial remission (PR) pre-ASCT and those with IgG isotypes serum samples had the highest rate of M-protein detection by miRAMM. miRAMM positivity at single time points (day 100, 6 months or 12 months) did not correlate with progression-free survival (PFS). In contrast, sCR patients who did not decrease their miRAMM M-protein intensities in serial measurements had shorter PFS than those whose miRAMM intensities decreased (median 17.9 months vs 51.6 months; *P*<0.0017). miRAMM M-protein is a more sensitive blood-based test than traditional M-protein tests and could cost effectively aid in serially monitoring complete remission for continue response or biochemical relapse.

## Introduction

As the number of effective therapies for treatment of multiple myeloma continues to increase, a greater proportion of patients are achieving deep therapeutic responses.^1, 2, 3^ Traditionally, serum and urine M-protein measurements by immunoelectrophoresis were sufficient to define treatment responses in 90% of cases.^4^ However, as more patients have deeper treatment responses, current M-protein detection methods are analytically incapable of monitoring patient deep response. Achieving and maintaining complete remission (CR) is important as it translates into longer periods of progression-free survival (PFS) and overall survival^5, 6^ but even among CR patients there is significant variability in the duration of overall survival.^7^ Therefore, in order to further stratify patients who achieve CR, more sensitive methods for detecting minimal residual disease (MRD) have been developed. To increase the analytical sensitivity, the focus has shifted towards analyzing the bone marrow (BM) for MRD. Two clinical BM MRD approaches have emerged: molecular-based methods detecting clonal variable diverse joining heavy chain rearrangements^8^ and multicolor flow cytometry^9^ for detecting malignant clonal plasma cells. BM molecular-based methods for MRD are >3 orders of magnitude more sensitive than serum M-protein detection by immunofixation (IFE) and free light chain (FLC).^10^ These more sensitive BM-based MRD methods are superior predictors of PFS. Unfortunately, patients classified as MRD negative continue to experience disease progression. It is possible that BM MRD methods may have sampling biases due to the patchy nature of BM infiltration.^11^ Therefore, there is still need for a test to monitor stringent complete response (sCR) patients for disease recurrence. Serial sampling using BM has drawbacks in terms of overall expense and patient discomfort. Therefore, a less invasive, cost effective test from blood may be useful in monitoring sCR patients.

Low-level quantification of a single immunoglobulin (Ig) M-protein clone in the midst of a polyclonal Ig background consisting of hundreds of thousand similar Ig molecules is both a daunting and intriguing proposition. Two mass spectrometric (MS) methods have been developed to extend M-protein quantification to lower levels. One method is based on the detection of unique tryptic peptides corresponding to the heavy and light chain variable region of an M-protein, which has been termed the ‘clonotypic’ peptide approach,^12, 13, 14^ and a second method, termed ‘miRAMM’ (monoclonal Ig rapid accurate mass measurements), which is based on identification of the M-protein from the accurate molecular mass of light chain component.^15^ Both methods take advantage of the fact that each M-protein undergoes a specific recombination event generating a unique variable domain and thus a unique molecular mass. Both methods provide accurate quantitative information with limits of detection that are 100 times lower than that of IFE.^14, 15^ The improvements in detection limits arises from superior resolution of accurate mass measurements to separate the M-protein from the polyclonal background in comparison with electrophoretic protein charge measurements. The miRAMM method has the intrinsic advantage of simplicity allowing for adaption to the clinical lab with shorter turnaround times than the clonotypic method. The clonotypic approach, however, is less dependent on the M-protein being expressed above the polyclonal background but is more prone to interference by low-level complementary determining region tryptic peptides from the endogenous polyclonal background as the M-protein level decreases.

The miRAMM method original described using liquid chromatography (LC)-coupled electrospray ionization-time-of-flight (TOF)-based MS platforms has also been adapted to a lower cost chromatography-free matrix assisted laser desorption/ionization (MALDI)-TOF MS platform with performance properties suited for the high volume clinical lab.^16^ Analytical times are rapid (<1 minute per patient) and when combined with five separate immunoprecipitations for IgG, IgA, IgM, κ and λ,the method can detect, quantitate and isotype M-proteins. The analytical equipment (Bruker, Microflex MALDI-TOF, Billerica, MA, USA) has also gained acceptance in the clinical lab for identification of bacteria. Recent application of MALDI-TOF MS method to patients with established clinical diagnosis demonstrated that MALDI-TOF MS had comparable clinical sensitivity with protein electrophoresis/IFE/FLC methods.^17^ However, although MALDI-TOF MS methods are quick and robust, electrospray ionization-TOF instruments provide superior resolving power and mass accuracy enabling a lower limit of detection. Thus, electrospray ionization-TOF miRAMM is better suited for quantitating low level M-proteins and resolving potential t-mAb drug interferences.

In this study, we have assessed the ability of a high sensitivity MS-based miRAMM method to extend the analytical range of traditional M-protein measurements in patients who in patients who achieve stringent CR (sCR) post autologous stem cell transplant (ASCT).

## Materials and methods

### Nanobody enrichment

Serum Ig enrichment was performed using a 50:50 mix of camelid-derived nanobodies directed against the LC constant domains of κ and λ (Thermo Fisher Scientific PN: 084910 and 083310), Life Technologies, Carlsbad, CA USA. Briefly, 10 μl of beads were incubated with 20 μl of serum diluted into 180 μl of phosphate-buffered saline for 45 min at ambient temperature. Subsequently, the supernatant was removed and the beads were washed three times with 200 μl of phosphate-buffered saline and then twice with 200 μl of water. Samples were eluted with 20 μl of 5% acetic acid containing 50 mM tris [2-carboxyethyl] phosphine, to disassociate Igs into separated LC and heavy chain components.

### Liquid chromatography

An Eksigent Ekspert 200 microLC (Foster City, CA, USA) was used to separate Ig LCs before ionization and detection. The mobile phases included an aqueous phase A (100% water+0.1% formic acid) and an organic phase B (90% acetonitrile+10% isopropanol+0.1% formic acid). Two microlitres of each bead elution was injected per analysis onto a Poroshell 300SB-C3 column (1.0 mm × 75 mm) with a 5 μm particle size placed in a 60 °C column heater. The gradient used has been described previously and the flow rate was 25 μl/min.^15^

### Mass spectrometry

A SCIEX TripleTOF 5600 quadrupole TOF MS using electrospray ionization in positive ion mode was used for miRAMM analysis. Source conditions have been described previously.^15^ Data analysis was performed using Analyst TF v1.6 and PeakView ver. 2.2 (AbSciex, Framingham, MA, USA). The mass spectra of the multiply charged LC ions were deconvoluted to accurate molecular mass using the Bio Tool Kit ver. 2.2 plug-in software (Asb Sciex, Framingham, MA, USA). The retention time of the monoclonal LC in each pre-treatment patient sample was tracked using PeakView. Subsequent detection of residual monoclonal LCs in post-treatment samples was performed by searching for the presence of a monoclonal LC above the polyclonal background with a molecular mass within ±1 Da of pre-treatment monoclonal LC with a retention time within 0.5 min of that identified in the pre-treatment sample. The LC signal intensity was recorded as counts per seconds. The instrument was calibrated every five samples using the automated calibrant delivery system. Mass measurement accuracy was estimated to be 15 p.p.m. over the course of the analysis.

### Patient samples

The Mayo Foundation Institutional Review Board approved this retrospective study. All patients gave written informed consent to have their medical records reviewed. Patient selection criteria included: availability of a serum sample within 30 days of initial diagnosis at Mayo Clinic Rochester from 2005 to 2012 and between 3 and 12 months post-ASCT, and attainment of a sCR using serum and urine IFE, serum Ig FLC and BM 6-color multicolor flow cytometry with a sensitivity of 10^−4^ to 10^−5^ within 120 days of ASCT. Thirty patients from the Mayo Clinic dysproteinemia sample bank fulfilled these criteria. A subset of these patients with durable CR had two stored serum samples in this 3–12 months interval.

## Results

**Figure 1:**
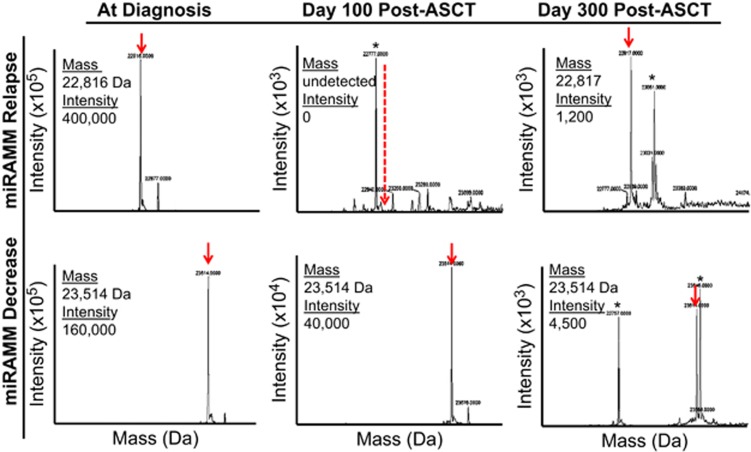
A deepening miRAMM response is suggestive of longer PFS in patients in sCR. Examples of miRAMM data. The top panel indicates a patient who had a miRAMM relapsed. At diagnosis the monoclonal LC mass signature was readily identifiable (red arrow). This mass signature was then assessed in subsequent post-treatment samples. At day 100, there is undetectable disease by miRAMM (dashed red arrow). The astrisk indicates a secondary oligoclonal response. However, ~200 days later there MS signature of this patients disease had re-emerged indicating a relapse (red arrow). The bottom panel indicates a patient with a deepening miRAMM response. Between diagnosis and 100 days post-ASCT, the MS signature for this patient decreased from an intensity of 160 000 to 40 000. Approximately 200 days later, this has continued to decline and is now at 4500.

The accurate molecular mass of the M-protein LC was readily identified (100% applicability) in all 30 diagnostic serum samples and served as a surrogate of M-protein identification in subsequent analysis (Figure 1). The use of a high-resolution MS capable of achieving a mass measurement accuracy (<15 p.p.m.) enabled the distinction of any LC with at least a 1 Da difference in mass. This mass accuracy was essential for evaluating post-ASCT serum samples as it was common for these samples to contain several ‘M-spikes’ in the LC mass distribution (Figure 1). Thus, to establish an M-spike in a post-ASCT sample as related to the original M-protein, the LC mass had to match the original peak within ±1 Da and the LC retention time within ±30 s of the diagnostic sample.

As per selection criteria for this study, all patients were documented in the electronic medical record to have achieved sCR at day 100 using conventional established testing criteria.^10^ All post-ASCT serum samples evaluated in this study were negative by serum protein electrophoresis, IFE and FLC assay. Sixteen of 30 patients had samples available for miRAMM testing collected when sCR was established at day 100 post-ASCT (±9 days). Of these samples, miRAMM identified the presence of the M-protein in 81% (13 of 16) of the patients (Table 1a). The three, day 100 miRAMM-negative cases were all IgA and were negative by serum protein electrophoresis pre-ASCT; surprisingly, these patients did not have prolonged PFS in comparison to the cohort. Twenty-five patients had serum samples available for testing by miRAMM that were collected between 6 and 12 months post-ASCT where sCR was maintained. Of these samples, 60% (15 of 25) were positive by miRAMM for M-protein (Table 1b). Patients who achieved CR pre-ASCT were more likely to be miRAMM negative 6–12 months post-ASCT as compared with those who did not (67% (4 of 6) and 32% (6 of 19), respectively). At these later time points, IgG and IgA isotypes were equally likely to be miRAMM negative (38% (6 of 16) versus 44% (4 out of 9)). In this small data set, single time points (day 100 or 6–12 months) miRAMM M-protein positivity status did not predict for better PFS.

The data were then re-examined in regards to the change in M-protein intensity between the two time points (Table 1c). Eleven^11^ patients had serum samples available at two different sampling dates (day 100 plus ~6–12 months) during which they remained in sCR. A rate of change in M-protein intensity was calculated by dividing the change in miRAMM intensity by the time interval between measurements in days. A continuing decrease in the miRAMM M-protein intensity (a positive rate of change) over time would be expected to correlate with a longer PFS, whereas failure to decrease or increasing intensities (a negative rate of change) should correlate with a shorter PFS. Of the 11 patients, 3 had converted to miRAMM-negative status at the second sampling, 1 had maintained miRAMM-negative status, 4 had a further reduction in miRAMM M-protein intensity (>40% from day 100 to the second sampling), 2 miRAMM M-proteins of stable intensity and 1 converted from miRAMM negative to miRAMM positive. The eight patients with continued miRAMM negativity or decreasing miRAMM M-protein intensity had longer PFS compared with those who had stable or increasing miRAMM M-protein intensity (51.6 months vs 17.9 months, respectively *P*<0.0017).

## Discussion

The data from this sCR cohort support previous studies, indicating that miRAMM is a more analytically sensitive method to detect M-proteins than electrophoresis.^15^ Although being more sensitive, the detection of M-protein at a single time point did not correlate with PFS for this relatively small non-homogeneous cohort. A confounding factor to in our data is the half-life of M-protein in blood (average 21–25 days for IgG and 7–14 days for IgA), which would cause a time lag between tumor lysis and M-protein decrease; an effect which becomes more pronounce at lower total Ig concentrations.^18^ Patients with IgA M-proteins and lower M-proteins concentrations pre-ASCT would need a shorter time interval to clear circulating M-protein from blood than IgG M-proteins with high levels pre-ASCT. This was supported by the day 100 results in which the three miRAMM-negative patients were all IgA patients (shorter half-life than IgG) with non-quantifiable M-protein pre-ASCT. By ~12 months, IgA and IgG were equally as likely to be miRAMM negative. Therefore, the timing of the evaluation by this method, or any sensitive blood method, needs to be carefully considered. Although Ig recycling can delay the clearance of the M-protein, it cannot cause the M-protein to increase or remain constant. Hence, the change in serial miRAMM M-protein measurements can aid in detecting active M-protein production. Thus, the finding of this study demonstrating that increases in the miRAMM M-protein intensity were correlated with shorter PFS is not surprising. miRAMM is well suited for serial sampling due to its cost-effective, less-invasive nature in comparison with BM aspiration. Further work in this area should focus on defining the biological and analytic variability of low level miRAMM M-proteins so that the clinical significance of changes in miRAMM M-protein intensity can be accurately defined. The results of this study highlight the recent recommendations from the IMWG consensus criteria for response and MRD in multiple myeloma.^10^ In their recommendation, the group states that the development of peripheral blood-based monitoring should be the ultimate goal as it would allow for serial sampling without the trauma of repeated BM aspirations and assure complete eradication of the tumor by assessment of the extramedullary compartment which is not evaluated by BM biopsy.

BM MRD status provides potentially important information not achievable by this method such as tumor clone evolution, cellular immune system reconstitution and direct detection of plasma cells. BM MRD is also not confounded by Ig half-lives. Hence, we do not see this method as substitute for BM MRD but rather as method to follow CR patients or as a companion method to accurately time BM MRD measurements. We acknowledge that miRAMM M-protein measurements may be subject to same limitations as other M-protein measurements such as in non-secretory myeloma. Other limitations of this study include a small sample size, limited follow-up, and inclusion of only patients with intact M-proteins rather than including patients with Bence Jones myeloma. Despite these limitations, our study provides compelling preliminary evidence that this simple MS-based test can serve as an assay that extends the current value of monitoring M-proteins beyond that of FLC, serum and urine IFE, and that serial sampling of patients can provide information on active M-protein production. Future studies evaluating the miRAMM method should also consider serial sampling with absolute quantification by miRAMM as opposed to the relative concentrations.

## Figures and Tables

**Table 1:** Patient characteristics and miRAMM results

*a. sCR patients tested at Day 100 post-ASCT in order of increasing miRAMM M-protein intensity*										
*Age (years)*	*Sex*	*FISH risk*	*Isotype*	*ISS*	*Pre-ASCT M-spike, g/dl*	*Induction response*	*Maintenance therapy*	*Days post-ASCT*	*miRAMM intensity (cps)*	*PFS (months)*
65	M	Intermediate	AK	3	Neg	CR	Yes	103	0	28
49	M	NA	AL	2	Neg	CR	No	105	0	15
67	F	Standard	AL	2	Neg	VGPR	No	98	0	19
61	M	NA	GL	1	0.6	PR	No	102	6700	45^a^
56	F	NA	AL	1	0.2	VGPR	No	103	10 000	10
44	M	Standard	GL	1	0.6	PR	No	102	10 300	18
59	F	Intermediate	AL	3	0.3	PR	Yes	102	10 900	60^a^
73	F	NA	AK	1	Neg	VGPR	No	91	24 000	93^a^
58	F	NA	GK	1	Neg	VGPR	No	100	24 600	38
61	M	High	AK	2	0.3	PR	No	96	32 300	5
67	M	NA	GK/AK	2	Neg	VGPR	Yes	105	32 900	61^a^
75	F	High	GK	2	0.5	PR	Yes	103	40 000	62^a^
58	M	Standard	GL	1	2.2	SD	No	102	49 000	60^a^
71	M	Standard	AK	3	Neg	VGPR	No	99	76 100	23^a^
64	F	Intermediate	GL	1	0.4	VGPR	No	101	90 400	178
71	M	NA	GK	3	Neg	CR	Yes	106	110 000	75^a^

*b. sCR patients tested at 6–12 months post-ASCT in order of increasing miRAMM M-protein intensity*										
*Age (years)*	*Sex*	*FISH risk*	*Isotype*	*ISS*	*Pre-ASCT M-spike, g/dl*	*Induction response*	*Maintenance therapy*	*Days post-ASCT*	*miRAMM intensity*	*PFS (months)*
49	M	NA	AL	2	Neg	CR	No	364	0	15
71	M	Standard	AK	3	Neg	VGPR	No	323	0	23^a^
67	M	NA	GK/AK	2	Neg	VGPR	Yes	364	0	61^a^
58	F	NA	GK	1	Neg	VGPR	No	346	0	38
55	F	NA	AK	2	Neg	PR	No	355	0	66
64	M	NA	GL	2	Neg	CR	Yes	300	0	34
65	M	Standard	AL	1	Neg	CR	No	353	0	39^a^
69	M	NA	GL	1	0.5	PR	No	246	0	29
51	F	NA	GK	3	Neg	CR	No	307	0	33
56	F	NA	GL	2	1.1	SD	No	194	0	137
62	F	High	AK	1	Neg	VGPR	No	280	800	25
67	F	Standard	AL	2	Neg	VGPR	No	278	1200	19
61	M	NA	GL	1	0.6	PR	No	370	2400	45^a^
41	M	NA	GK	1	Neg	VGPR	No	173	4400	86
75	F	High	GK	2	0.5	PR	Yes	194	4500	62^a^
58	M	Standard	GL	1	2.2	SD	No	305	4800	60^a^
56	F	NA	AL	1	0.2	VGPR	No	297	9500	10
44	M	Standard	GL	1	0.6	PR	No	362	12 100	18
73	F	NA	AK	1	Neg	VGPR	No	350	12 800	93^a^
41	F	NA	GL	3	Neg	CR	No	179	19 200	75^a^
66	F	Standard	AL	3	Neg	VGPR	Yes	168	25 700	55^a^
61	M	High	GK	2	0.9	PR	No	189	26 400	36
50	M	High	GL	1	0.3	PR	Yes	357	28 000	15
66	M	NA	GK	2	0.4	PR	No	189	89 500	31
71	M	Standard	GK	3	Neg	CR	Yes	202	108 000	42

*c. sCR patients with miRAMM testing at Day 100 and at 6–12 months post-ASCT in order of decrease rate of change*^b^ *of M-protein intensity*													
*Age (years)*	*Sex*	*FISH risk*	*Isotype*	*ISS*	*Pre-ASCT M-spike, g/dl*	*Induction response*	*Maintenance therapy*	*Days post-ASCT*	*Day 100 miRAMM intensity*	*Days post-ASCT*	*6–12 Months miRAMM intensity*	*Rate of change*	*PFS (months)*
49	M	NA	AL	2	Neg	CR	No	105	0	364	0	NA	15
75	F	High	GK	2	0.5	PR	Yes	103	40 000	194	4500	400	62^a^
71	M	Standard	AK	3	Neg	VGPR	No	99	76 100	323	0	300	23^a^
58	M	Standard	GL	1	2.2	SD	No	102	49 000	305	4800	200	60^a^
67	M	NA	GK/AK	2	Neg	VGPR	Yes	105	32 900	364	0	100	61^a^
58	F	NA	GK	1	Neg	VGPR	No	100	24 600	346	0	100	38
73	F	NA	AK	1	Neg	VGPR	No	91	24 000	350	12 800	40	93^a^
61	M	NA	GL	1	0.6	PR	No	102	6700	370	2400	20	45^a^
56	F	NA	AL	1	0.2	VGPR	No	103	10 000	297	9500	0	10
67	F	Standard	AL	2	Neg	VGPR	No	98	0	278	1100	-10	19
44	M	Standard	GL	1	0.6	PR	No	102	10 300	362	12 100	-10	18

Abbreviations: AK, IgA kappa; AL, IgA lambda; ASCT, autologous stem cell transplant; cps, counts per seconds; CR, complete remission; F, female; FISH, fluorescence *in situ* hybridization; GK, IgG kappa; GL, IgG lambda; ISS, International Staging System; M, male; miRAMM, monoclonal immunoglobulin rapid accurate mass measurement; NA, not applicable; Neg, negative; PFS, progression-free survival; PR, partial remission; sCR, stringent complete response; SD, stable diseas; VGPR, very good partial remission.

a Censored data, these patients remain disease-free at the time of writing this manuscript.

b A rate of change in miRAMM M-protein intensity was calculated by dividing the change in miRAMM intensity by the time interval between measurements in days.
